# Diazotrophs are overlooked contributors to carbon and nitrogen export to the deep ocean

**DOI:** 10.1038/s41396-022-01319-3

**Published:** 2022-09-26

**Authors:** Sophie Bonnet, Mar Benavides, Frédéric A. C. Le Moigne, Mercedes Camps, Antoine Torremocha, Olivier Grosso, Céline Dimier, Dina Spungin, Ilana Berman-Frank, Laurence Garczarek, Francisco M. Cornejo-Castillo

**Affiliations:** 1 Aix Marseille University, Université de Toulon, CNRS, IRD, MIO UM 110, 13288 Marseille, France; 2 Turing Center for Living Systems, Aix-Marseille University, 13009 Marseille, France; 3 LEMAR, Laboratoire des sciences de l’environnement marin, UMR6539, CNRS, UBO, IFREMER, IRD, 29280 Plouzané, Technopôle Brest-Iroise France; 4 Laboratoire d’Océanographie de Villefranche (LOV), Sorbonne Université, CNRS, 06230 Villefranche sur mer, France; 5 University of Haifa, The Leon H. Charney School of Marine Sciences, Haifa, 3498838 Israel; 6 Sorbonne Université, CNRS, UMR 7144 Adaptation and Diversity in the Marine Environment (AD2M), Station Biologique de Roscoff (SBR), Roscoff, France; 7 Institut de Ciènces del Mar (ICM-CSIC), E08003 Barcelona, Spain

**Keywords:** Biogeochemistry, Microbiology

## Abstract

Diazotrophs are widespread microorganisms that alleviate nitrogen limitation in 60% of our oceans, thereby regulating marine productivity. Yet, the group-specific contribution of diazotrophs to organic matter export has not been quantified, which so far has impeded an accurate assessment of their impact on the biological carbon pump. Here, we examine the fate of five groups of globally-distributed diazotrophs by using an original combination of mesopelagic particle sampling devices across the subtropical South Pacific Ocean. We demonstrate that cyanobacterial and non-cyanobacterial diazotrophs are exported down to 1000 m depth. Surprisingly, group-specific export turnover rates point to a more efficient export of small unicellular cyanobacterial diazotrophs (UCYN) relative to the larger and filamentous *Trichodesmium*. Phycoerythrin-containing UCYN-B and UCYN-C-like cells were recurrently found embedded in large (>50 µm) organic aggregates or organized into clusters of tens to hundreds of cells linked by an extracellular matrix, presumably facilitating their export. Beyond the South Pacific, our data are supported by analysis of the *Tara* Oceans metagenomes collected in other ocean basins, extending the scope of our results globally. We show that, when diazotrophs are found in the euphotic zone, they are also systematically present in mesopelagic waters, suggesting their transport to the deep ocean. We thus conclude that diazotrophs are a significant part of the carbon sequestered in the deep ocean and, therefore, they need to be accounted in regional and global estimates of export.

## Introduction

Nitrogen (N) availability limits primary productivity throughout much of the surface low-latitude ocean [1]. In such nitrogen (N)-limited waters, microbial dinitrogen (N_2_) fixation by diazotrophic plankton provides the major source of new N to the surface ocean [2], maintaining ocean fertility and, on appropriate timescales, is equivalent to export production to the deep ocean [3]. However, the fate of this production remains obscure [4, 5]. Currently, there is no consensus as to whether diazotrophically fixed N is exported to the deep ocean or if it stimulates remineralization in surface waters. An increasing number of studies have shown that diazotroph-derived N is quickly translocated to non-diazotrophic plankton such as diatoms [6, 7], which eventually contributes to secondary export of organic matter out of the photic zone. Yet, except for Diatom-Diazotroph Associations (DDAs) [8], the direct gravitational settling of diazotrophs themselves to the deep ocean has rarely been quantified.

Diazotrophs may associate with sinking particles and contribute to direct export by different mechanisms. The most direct ones include gravitational settling of individual cells/filaments or aggregates. According to the Stokes’ law, particle sinking velocity scales with the square of particle size. Therefore, large particles should sink faster and are more likely to reach the deep ocean before being remineralized by bacteria [9]. However, past studies have revealed the importance of small (<2 µm) phytoplankton (including the non-diazotrophic *Synechococcus* and *Prochlorococcus*) in carbon export, especially in oligotrophic ocean regions [10, 11]. Aggregation is one of the crucial steps for the transport of these small plankton, which could export particulate organic carbon (POC) in similar proportion to their production in surface waters [11]. Diazotrophs have diverse morphologies and their size spans several orders of magnitude. Some types such as the free-living unicellular diazotrophic cyanobacteria (UCYN from groups B and C) are small (2–8 μm in diameter), while others such as *Trichodesmium sp*. are filamentous and can form large-sized colonies (>100–1000 µm). In addition, some diazotrophs live in symbiosis with calcified (UCYN-A, ~1 μm) or silicified eukaryotes (*Richelia* sp., *Calothrix* sp., >20 µm, forming DDAs). These dense biominerals may provide ballast enhancing the downward export of these symbioses into the deep ocean. Therefore, the presence of different diazotrophs in surface waters may result in drastically different export fluxes. Yet, to date, no field observations have explored how efficiently diazotrophs are exported, and if some are exported more efficiently than others, which prevents robust predictions of how diazotrophs contribute to the biological carbon pump.

Thanks to their inherently ballasted character, DDAs are well known to contribute to particulate matter export [12] and are involved in seasonal peaks of POC export to the deep sea (4000 m) in the North Pacific subtropical gyre [8]. *Trichodesmium*, one of the major contributors to global N_2_ fixation [13], is thought to have a limited export capacity and to be preferentially remineralized in the surface layers due to the presence of gas vesicles providing them buoyancy [4, 14, 15]. Yet, some studies have reported its presence in sediment traps material in the Kuroshio Current [16], the tropical North [17] and South Pacific Oceans [18]. Intact filaments and colonies of *Trichodesmium* sp. have also been reported as deep as 3000–4000 m in the tropical Atlantic, Pacific and Indian Oceans [19, 20], but their contribution to organic matter export has yet to be quantified.

Theoretically, UCYN may not contribute significantly to POC export fluxes due to their small size. Yet, Berthelot et al. [21] reported that primary production supported by UCYN was twice as efficient in promoting POC export than production supported by DDAs. Recent studies confirmed the presence of UCYN-B in sinking particles in the meso- and bathypelagic ocean [18, 22], but the export of such small particles (1–8 µm) remains enigmatic. Finally, Farnelid et al. [23] observed *nifH* gene sequences in exported material (150 m) in the North Pacific subtropical gyre and found that all the diazotroph groups above as well as non-cyanobacterial diazotrophs were present in the samples.

Taken together, these studies suggest that all diazotroph groups, small or large, free-living or symbiotic, ballasted or not, have been detected below the photic layer, raising the question of their potential impact on the biological carbon pump. A detailed examination relating diazotroph types to the magnitude of downward biogenic elements fluxes is needed to refine our understanding of their role in the mechanisms controlling the export of organic matter in the ocean. This is a pressing question as diazotrophs have recently been identified as key drivers influencing the response of future marine net primary productivity to climate change [24].

**Fig. 1:**
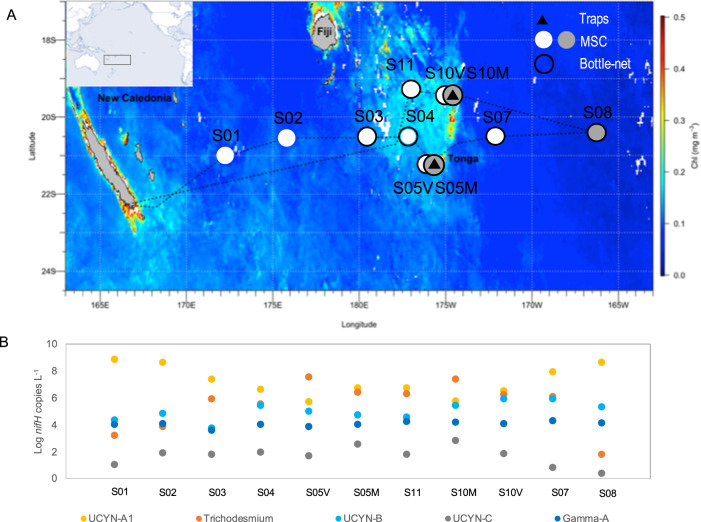
Sampling stations and surface diazotroph abundances. **A** Satellite-derived surface chlorophyll *a* concentrations during the GPpr14 cruise (1 November-6 December 2019) (MODIS Aqua, 4 km, 8-days composite, level 3 product). Black triangles correspond to stations where surface-tethered drifting sediment traps were deployed (170 m, 270 m, 1000 m). Grey dots correspond to stations where Marine Snow Catcher (MSC) casts were performed at three depths (see Methods), and white dots to MSC casts performed at one depth (200 m). Black circles correspond to stations where the bottlenet profiles were performed between 2000 m and 200 m. **B** Abundances (Log10 *nifH* gene copies L^−1^) of the five *nifH* phylotypes targeted over the transect (dots represent abundances averaged over the photic layer, ~0–100 m).

Here, we examine the group-specific fate of diazotrophs in the mesopelagic ocean. We used an innovative approach consisting of the combined deployment of surface-tethered drifting sediment traps, a Marine Snow Catcher (MSC), and a Bottle-net, in which we performed *nifH* gene sequencing and quantitative PCR on major diazotroph groups across the subtropical South Pacific Ocean (Fig. 1A) in parallel with export flux quantification. We show that all globally significant N_2_-fixing cyanobacteria and non-cyanobacterial diazotrophs are systematically present in sinking particles down to 1000 m. Small size UCYN (1–8 µm) are exported more efficiently than large filamentous diazotrophs under the form of large (>50 µm) aggregates linked by an extracellular matrix. Globally, our analysis of the *Tara* Oceans metagenomes confirms that diazotrophs are always detected in mesopelagic waters when present in surface waters, potentially revealing that the transport of diazotrophs to the deep ocean is an important pathway for diazotroph-derived export production, influencing the ability of our ocean to sequester carbon.

## Materials and methods

Samples were collected during the GEOTRACES GPpr14 expedition (10.17600/18000884) in the subtropical South Pacific Ocean (Fig. 1) in austral summer (Nov.-Dec. 2019).

### Photic layer sampling

Vertical 0–150 m depth profiles were performed at all stations using a trace metal clean titanium rosette of Go-Flo bottles equipped with a fluorometer and temperature, conductivity and oxygen sensors. Seawater samples were collected from 5 depths (75, 50, 20, 10, 1% surface irradiance levels) to quantify the stock of major groups of diazotrophs in the photic layer by quantitative PCR (qPCR) as described below.

### Sediment traps deployment and sample analyses

A surface tethered mooring line (~1000 m long) was deployed at stations S05M (5 days) and S10M (4 days). The line was equipped with sediment traps (KC Denmark^®^) at 3 depths: 170 m, 270 m and 1000 m. Each trap was composed of four particle interceptor tubes (PITs) mounted on a cross frame (collecting area of 0.0085 m^2^, aspect ratio of 6.7) filled with 0.2 μm filtered seawater with added saline brine (50 g L^−1^). Two tubes were used for this study: one for biogeochemical analyses (also filled with borate-buffered formalin (5%) [25]), and one for microbiological analyses. After recovery, the density gradient was visually verified, and the PITs were allowed to settle for 2 h before the supernatant seawater was carefully removed with a peristaltic pump. The remaining water containing the sinking material was transferred to a chlorhydric acid-washed container, while being screened with a 500 μm mesh to remove swimmers [26]. Subsequently, samples were split into 12 aliquots. A triplicate set of aliquots were filtered onto 25 mm diameter Supor filters for *nifH* sequencing and *nifH* qPCR as described below. Another set of triplicate samples were filtered onto 25 mm diameter combusted (4 h, 450 °C) glass microfiber filters (Whatman GF/F), which were subsequently dried for 24 h at 60 °C, pelleted and from which particulate N (PON) and C (POC) were analyzed by EA-IRMS (Elemental Analyzer-Isotope Ratio Mass Spectrometry) using an Integra 2 (Sercon) mass spectrometer. Lastly, a triplicate set of aliquots was filtered on GF/F filters for further pigment analyses [27], and another set filtered for microscopic analyses (see below for methods).

### MSC deployment

Suspended and sinking particles were sampled using a MSC at 3 depths at the mooring stations S05M and S10M (170 m, 270 m and 1000 m), and at 200 m, 400 m and 1000 m at S8. Additionally, the MSC was deployed at 200 m at S01, S02, S03, S04, S05V, S07, S10V and S11 (Fig. 1). The MSC is a large volume water sampler (100 L) that collects sinking particles with minimal turbulent agitation [28]. Upon recovery, the MSC is conventionally placed on deck for 2 h while any particles present settle onto the base of the bottom 7 L chamber [29]. The fraction containing fast-sinking particles was thereby collected in a dedicated plate at the bottom of the MSC (280 to 310 mL), the one containing slow sinking particles (1.2 L) was collected from the bottom chamber, and the non-sinking (or suspended) fraction (4 L) was collected from the upper part of the MSC [29]. Each fraction was filtered for PON and POC quantification, microscopic observations, *nifH* sequencing and *nifH* qPCR as for the traps. Transparent Exopolymeric Particles (TEP) concentrations were also quantified in each fraction according to Passow and Alldredge (1995) [30]. The total amount of TEP-C was calculated by multiplying the volumetric TEP concentrations (in µg equivalent gum xanthan per liter) by the conversion factor of 0.63 µg C µg^−1^ xanthan gum [31]. We used the formula described in Riley et al., (2012) [29] to obtain values associated with fast or slow- sinking particles only, quantitatively removing the contribution of other fractions.

### Bottle-net deployment

Vertical 2000–200 m profiles were done using a bottle-net mounted on the CTD rosette frame at stations S03, S04, S05M, S07, S08, S10M, S11 and S12. The bottle-net consists of a 20 μm conical plankton net housed in a cylindrical PVC pipe [19]. The top cover is opened at the desired bottom depth (2000 m) of the tow, remains opened during the ascent of the rosette, and closed again at the upper depth (200 m) of the water column to be sampled. This results in one integrated sample of 200 m to 2000 m per deployment. Once on deck, the bottle-nets were gently rinsed with filtered seawater before retrieving the sample from the collector, that was processed for *nifH* qPCR. Sampled volume was estimated as the product between the cross-sectional area of the mouth of the bottle-Net (7.5 cm, aspect ratio of 4) and the vertical distance covered by the device from the start of the ascension to the closure of the top cover (1800 m). Blank casts were performed with the bottle-net closed during the entire cast to assess for potential contaminations, and blanks were subtracted.

### *nifH* gene sequencing and bioinformatics

The *nifH* gene was sequenced from a total of 71 samples (18 samples from sediment traps and 53 MSC samples). DNA was extracted using the DNeasy Plant Mini Kit (Qiagen, Courtaboeuf, France) with additional freeze-thaw bead beating and proteinase K steps before the kit purification [32]. Triplicate nested PCR reactions were conducted using the degenerate primers nifH3 (ATRTTRTTNGCNGCRTA) and nifH4 (TTYTAYGGNAARGGNGG) in the first PCR followed by a second amplification with nifH1 (TGYGAYCCNAARGCNGA) and nifH2 (ADNGCCATCATYTCNCC) primers [33]. The PCR mix was composed of 5 μL of 5X MyTaq red PCR buffer (Bioline), 1.25 μL of 25 mM MgCl_2_, 0.5 μl of 20 μM forward and reverse primers, 0.25 μL Platinum Taq and 5 μL of DNA extract (1 μL on second round). The reaction volume was adjusted to 25 μL with PCR grade water. Triplicate PCR products were pooled and purified using the Geneclean Turbo kit (MP Biomedicals). Partial adapters were added by ligation at the sequencing facility (Genewiz) and llumina MiSeq 2 × 300 paired end sequenced. Demultiplexed paired-end sequences were dereplicated, denoised, assembled and chimeras discarded using the DADA2 pipeline [34]. 10,923 to 34,844 reads were obtained per sample (23,361 reads/sample on average). In total, >1.8 millions of high quality *nifH* sequences were obtained resulting in 1566 ASVs (Amplicon Sequence Variants). ASVs were annotated down to the genus level using a DADA2 formatted *nifH* gene database (https://github.com/moyn413/nifHdada2). ASVs accounting for 1% or more of the reads for at least one of the samples were grouped into 14 genera according to the database (Table S1), and those sequences not identified to the genus level were grouped as “rare”, although they represented 37 phylogenetic groups (clustered at 95% of nucleotide identity). The *nifH* gene was successfully amplified from all samples.

### Abundance of diazotrophs and contribution to N export fluxes

The abundance of diazotrophs was determined using TaqMan qPCR assays and previously published primer-probe sets for *Trichodesmium*, UCYN-A1, UCYN-B, UCYN-C and γ-24474A11 (hereafter referred to as Gamma-A) targeting the *nifH* gene [35, 36]. The qPCR was run in 25 µL reactions consisting of 12.25 µL TaqMan PCR Master Mix (Applied Biosystems, Villebon Sur Yvette, France), 1 µL of the forward and reverse primers at 10 µM (HPLC purified, Eurofins, Nantes, France), 0.25 µL probe at 10 µM, 8.25 µL PCR grade water, 0.25 µL bovine serum albumin at 10.08 µg µL^−1^, and 2 µL standard or template sample. The qPCR program was run on a CFX96 Real-Time System thermal cycler (BioRad, Marnes-la-Coquette, France) and consisted of 2 min at 50 °C, 10 min at 95 °C continued by 45 cycles of 15 s at 95 °C and 1 min at 64 °C. The annealing temperature was changed to 60 °C for UCYN-A1 qPCR runs [36]. Standards were generated from custom produced plasmids with the target insert of interest (GENEWIZ Co. Ltd., Suzhou, China). Plasmids were linearized by HindIII (Thermo Fisher Scientific) digestion, gel purified, and quantified using Picogreen. Each 96 well plate was run with a serial dilution (10^0^–10^7^ nifH copies reaction^−1^) of the appropriate standard in duplicates. Samples and no-template controls (NTCs) were run in triplicate. NTCs did not show any amplification. The efficiency was 98–113%. Inhibition tests were carried out on all samples and each primer-probe set by adding 2 µL of the 10^5^ copies standard to each sample. No inhibition was observed. The limit of detection and detected but not quantifiable limits were 1 and 8 gene copies per reaction, respectively.

The diazotroph turnover rate representing the fraction of surface diazotrophs exported out of the photic layer per day was calculated as follows: Turnover rate (d^−1^) = export flux (*nifH* copies m^−2^ d^−1^)/integrated abundance over the photic layer *(nifH* copies m^−2^). The proportion of PON collected by sediment traps attributed to diazotrophs was roughly estimated based on the product of qPCR-based abundances of each diazotroph phylotype, published POC content per cell [13] and C:N ratios (see details in Table S2). A potential pitfall when doing these calculations is that polyploidy has been reported in natural diazotroph populations [37–39], which may inflate their contribution to PON export. In order to take into account polyploidy, we quantified the abundance of *Trichodesmium* and UCYN-B (the two abundant groups that can be enumerated by microscopy) at 4 depths in the photic layer by using both qPCR and epifluorescence microscopy. The ratio between qPCR and microscopy for *Trichodesmium* was 12 (S05M) and 38 (S10M), i.e. in accordance with earlier field studied [38] and 9 (S05M) and 13 (S10M) for UCYN-B. These numbers were used as a proxy of polyploidy (number of nifH copies per cell) and we divided the qPCR abundances in the traps by these numbers.

### Particle imaging

Seawater samples from traps and MSC were filtered on 0.2 µm (for scanning electron microscopy, SEM) and 2 µm polycarbonate filters (for epifluorescence microscopy) at very low pressure to preserve the particle structure. For epifluorescence microscopy, filters were fixed with paraformaldehyde (2% prepared in filtered seawater) for 10 min at ambient temperature and stored at −80 °C until visualized using a Zeiss Axioplan (Zeiss, Jena, Germany) microscope fitted with a green (510–560 nm) excitation filter, which targeted the phycoerythrin-rich cells. UCYN were discriminated from other phycoerythrin-containing cyanobacteria (i.e. the picocyanobacteria *Synechococcus*, ~1 µm size) based on their larger size (>4 µm). For SEM, samples were fixed with 2.5% glutaraldehyde and 1.6% PFA for 1 h at room temperature. Filters were then rinsed twice in 0.2 µm filtered in seawater (15 min), rinsed in osmium (30 min), and rinsed thrice with filtered seawater to eliminate excess osmium. Filters were then passed through a series of ethanol drying solutions (50, 70, 95 and 100%, 10 min each), and a series of HDMS solutions (30, 50, 80 and 100%, 10 min each), before being air-dried, and stored at room temperature until visualized onshore using a Phenom-Pro benchtop scanning electron microscope at 10 kV.

### *Tara* Oceans sampling and read recruitments in metagenomes

23 metagenomes, collected from five stations along the *Tara* Oceans expedition transect and corresponding to a subset of the data presented in Karlusich et al., (2021) [37], were selected for this study since these stations were the only ones for which: i) metagenomic reads belonging to *nifH* gene sequences from distinct cyanobacterial diazotrophs were detected in surface waters and ii) metagenomes samples from both surface (5 m) and mesopelagic waters (200–1000 m) were collected and sequenced (Table S3). Briefly, the plankton were separated into discrete organismal size fractions using a serial filtration system [40] corresponding to picoplankton size (0.2–3 µm), nanoplankton (0.8–3 µm or 0.8–5 µm) and a ‘bulk’ size fraction corresponding to the fraction >0.8 µm or >3 µm. Metagenomes were sequenced as Illumina overlapping paired reads of 100–108 bp, which were merged and trimmed based on quality, resulting in 100–215 bp fragments [41]. Metagenomic reads were recruited against a database of 9 representative genomes of cyanobacterial diazotrophs (Table S3) using BLASTN (v2.9.0; (Altschul et al., 1990 [42]) with default parameters but limiting the results to one target sequence (--max_target_seqs 1) and keeping only results with an E-value below 1e-30 (-evalue 1e-30) and with a query coverage of, at least, 90% of the read length (-qcov_hsp_perc 90). Following the criteria proposed by Caro-Quintero & Konstantinidis [43] for metagenome-based prokaryotic genome classification, reads mapping to diazotroph genomes with a percentage of nucleotide identity equal or greater than 95% were taxonomically assigned to each diazotroph genome according to their best hit, except reads that mapped to the ribosomal operon of any of the diazotroph genomes that were filtered out. The number of reads recruited by each diazotroph genome was normalized to the sequencing depth of each sample. Only samples for which genome coverage was higher than 1% were taken into account for each organism.

## Results and discussion

### Surface conditions

The study region was characterized by typical oligotrophic waters (chlorophyll concentrations <0.15 µg L^−1^, Deep Chlorophyll Maximum (DCM) 100–180 m) east and west of Tonga, corresponding to the first group of stations S01, S02, S07 and S08 (Fig. 1A). The second group of stations (S03, S04, S05, S10, S11) was located in mesotrophic waters (chlorophyll > 0.15 µg L^−1^, DCM 70–90 m) in the vicinity of the Tonga archipelago (Fig. 1A). Surface (0–50 m) nitrate concentrations were consistently close or below the detection limit (0.05 µmol L^−1^) throughout the transect, while phosphate concentrations were typically 0.1 µmol L^−1^ at oligotrophic stations and depleted down to detection limit (0.05 µmol L^−1^) at mesotrophic stations, likely due to consumption by higher plankton stocks (Fig. S1). Seawater temperature ranged from 23.1 to 27.3 °C in the mixed layer (0–15 m to 0–60 m) determined according to de Boyer Montégut et al. (2004) [44] (Fig. S1).

The *nifH* abundances of key diazotroph groups indicated that UCYN-A1 were present at high abundances throughout the photic layer (~0–100 m) over most of the transect (average 3.4 × 10^6^ *nifH* gene copies L^−1^) (Fig. 1B). *Trichodesmium* was the second most abundant group, particularly in the vicinity of Tonga (average 6.8 × 10^6^ *nifH* gene copies L^−1^), followed by UCYN-B (3.9 × 10^5^ *nifH* gene copies L^−1^). UCYN-C and Gamma-A (a non-cyanobacterial diazotroph) were also detected throughout the transect albeit at lower abundances (10^2^ to 10^4^ *nifH* gene copies L^−1^).

### How efficient are different diazotrophs groups to export?

**Fig. 2:**
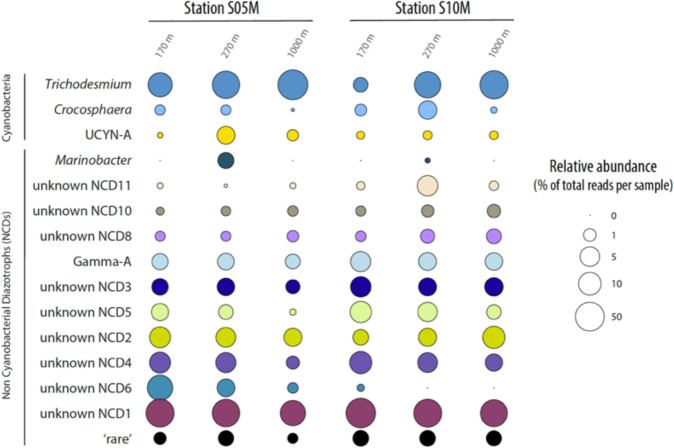
Relative abundance of diazotroph groups in sediment trap samples across depths (170 m, 270 m, 1000 m) at stations S05M and S10M. Diazotroph groups are defined as ASVs sharing more than 95% nucleotide identity. Diazotroph groups contributing less than 1% were pooled together and are shown as ‘rare’. Values are presented in log10 scale.

We first examined the *nifH* gene community composition of particles collected in drifting sediment traps located at 170 m, 270 m and 1000 m depth at stations S05M and S10M (Fig. 1A). On average over the three depths, 43 ± 23% and 31 ± 20% of the retrieved ASVs corresponded to cyanobacteria genera at stations S05M and S10M, respectively (Fig. 2). The most abundant genus was *Trichodesmium*, whose relative contribution increased towards deeper waters at both stations. ASVs related to *Crocosphaera watsonii* (UCYN-B) and *Candidatus* Atelocyanobacterium thalassa (UCYN-A) were also identified at lower relative contribution, and genera related to DDAs (*Richelia* spp., *Calothrix* spp.) were not represented among the ASVs. However, a recently reported marine cyanobacterial endosymbiont of diatoms closely related to the freshwater spheroid bodies of *Epithemia turgida* and *Rophalodia gibba* [37], accounted for a very small, but recurrent proportion (0.01–0.12%) of the reads at both stations. Besides autotrophic diazotrophs, 57 ± 22% and 69 ± 19% of ASVs were affiliated to non-cyanobacterial diazotrophs at stations S05M and S10M, respectively, including the Gammaproteobacteria Gamma-A and *Marinobacter* (Fig. 2). Interestingly, the ASV that contributed the most at both stations (ASV1, ‘unknown NCD1’; Fig. 2; Table S1), to the best of our knowledge, has never been found in any previous study.

**Fig. 3:**
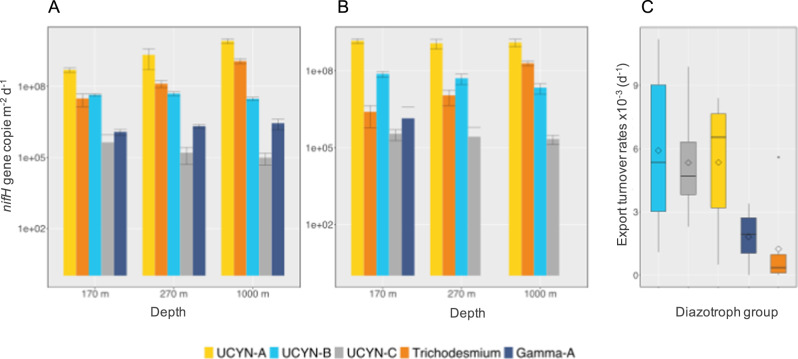
Quantification of diazotrophs in sediment traps. Export flux (*nifH* gene copies m^−2^ d^−1^) of the five diazotroph groups targeted by qPCR (UCYN-A1 symbiosis, UCYN-B, UCYN-C, *Trichodesmium* and Gamma-A) in sediment trap samples at 170 m, 270 m and 1000 m at **A** station S05M and **B** station S10M. Error bars represent strandard deviations from triplicate aliquotes analyzed in duplicates. **C** Export turnover rates (d^−1^)=export flux (*nifH* copies m^−2^ d^−1^)/integrated abundance over the photic layer *(nifH* copies m^−2^). The average export flux of the 3 sediment trap depths was used for the calculation.

High relative abundances in PCR-based libraries is not indicative of high absolute abundance, as often these sequence types dominate PCR libraries, although being present at quite low abundances in the environment [45]. Hence, to assess the export capacity of individual diazotroph groups, we quantified the abundance of five groups spanning different forms, sizes, lifestyle (symbiotic or not), using qPCR assays (UCYN-A1 symbiosis, UCYN-B, UCYN-C, *Trichodesmium* and Gamma-A; see Methods). DDAs were not quantified as they were almost not detected by microscopy nor by sequencing, and were probably rare at the sampled depths at the time of the cruise. The diazotroph assemblage exported to sediment traps generally reflected that of the photic layer, although some diazotrophs were exported more efficiently than others (Fig. 3A, B). The highest diazotroph export fluxes were measured for UCYN-A1 at both sites and all depths of traps deployment (5.0 ± 1.1 × 10^8^ to 8.5 ± 2.0 × 10^9^ *nifH* gene copies m^−2^d^−1^), followed either by UCYN-B (2.2 ± 0.9 × 10^7^ to 7.5 ± 1.4 × 10^7^ *nifH* gene copies m^−2^d^−1^) or *Trichodesmium* (2.5 ± 1.9 × 10^6^ to 1.2 ± 0.3 × 10^8^ *nifH* gene copies m^−2^d^−1^) depending on station and depth (Fig. 3A, B). Gamma-A and UCYN-C were also exported, albeit at lower rates (~10^5^ and ~10^6^ *nifH* gene copies m^−2^d^−1^, respectively).

Specific export turnover rates provide information on the rate at which diazotrophs are “lost” from the photic layer due to export (Turnover rate (d^−1^) = export flux/abundance in the photic layer). The range of calculated rates (0.7 ± 1.2 × 10^–5^ to 11.2 ± 1.9 × 10^−3^d^−1^) spans through two orders of magnitude and varied depending on the diazotroph group, depth and station considered. On average (over all stations and depths), the export turnover rate of UCYN (5.5 ± 3.1 × 10^−3^ d^−1^) was generally higher (by ca. four times) than that of *Trichodesmium* (1.3 ± 2.2 × 10^−3^ d^−1^) (Fig. 3C), suggesting that UCYN groups are more efficiently exported than the filamentous *Trichodesmium*. Among UCYN groups, the highest export turnover rate was measured for UCYN-B, followed by UCYN-C and UCYN-A1, although differences were not significant between groups (Mann-Whitney test, *p* < 0.05). The export turnover rate of Gamma-A (average 1.8 ± 1.5 × 10^−3^d^−1^) was intermediate between that of *Trichodesmium* and UCYN groups.

**Fig. 4:**
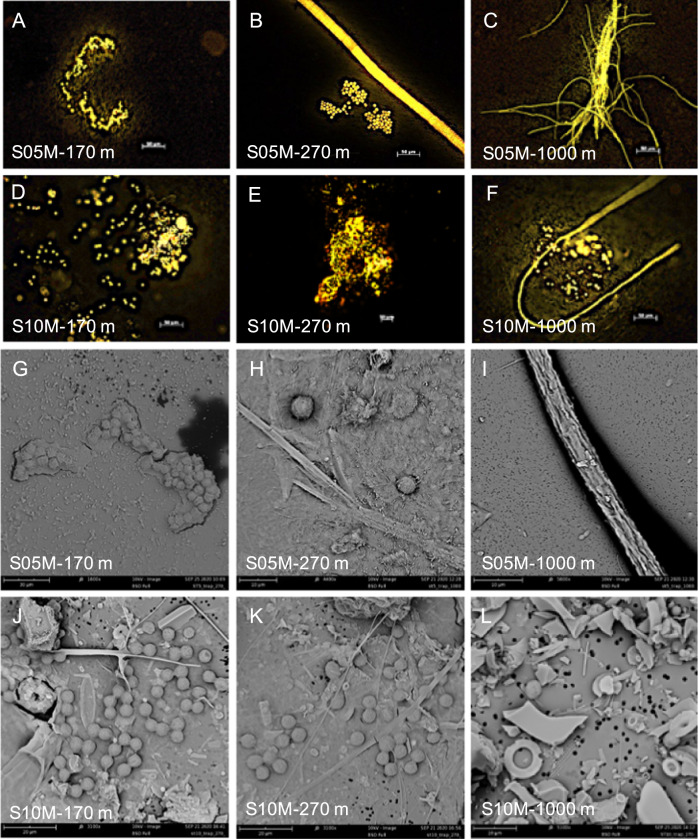
Microscopy images showing examples of phycoerythrin-containing UCYN-like cells and Trichodesmium in sediment trap samples collected at 170 m, 270 m, and 1000 m at stations S05M and S10M. **A**–**F** Images taken by epifluorescence microscopy (green excitation 510–560 nm, scale bar: 50 µm). **G**–**L** Images taken by scanning electron microscopy (SEM).

Epifluorescence microscopy confirmed the presence of phycoerythrin-containing UCYN-B and UCYN-C-like cells in sediment trap samples (size > 2 µm, i.e. easily distinguishable from picocyanobacteria such as *Synechococcus*) (Fig. 4A–F). SEM revealed that they were recurrently found embedded in large organic aggregates or organized into clusters of tens to hundreds of cells linked by an extracellular matrix (Fig. 4G–L), which was further confirmed by Alcian blue straining (Fig. S2). *Trichodesmium* was also observed in all samples, mostly as free filaments, but intact colonies were observed in sediment trap samples especially at 1000 m at both stations. Gamma-A and UCYN-A1 cannot be visualized by these techniques and are thus assessed solely on the basis of qPCR (above).

Pigment concentrations measured in sediment trap samples at both stations indicate that total pigment concentrations and diversity decreased with depth. Apart from the degradation pigments phaeophorbide and phaeophytin, zeaxanthin, a biomarker of cyanobacteria, predominated at all depths (Fig. S3). The Chlorophyll *a*: phaeopigments ratios were elevated (average 1.8, range 0.8–3.), indicative of fresh organic matter in sediment trap samples.

### The fate of diazotrophs in the mesopelagic ocean

**Fig. 5:**
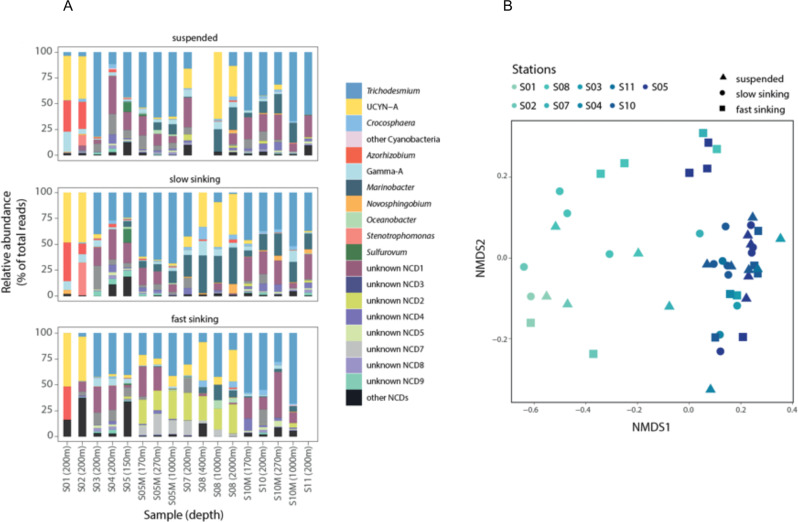
Diazotroph communities collected from Marine Snow Catcher (MSC) samples. **A** Relative contribution of diazotroph groups detected in different MSC fractions (suspended, fast sinking and slow sinking). As in Fig. 2, diazotroph groups are defined as ASVs sharing more than 95% nucleotide identity. Diazotroph groups contributing less than 0.5% on average across samples were pooled together and are shown as ‘other Cyanobacteria’ and ‘other NCDs’ if they were taxonomically classified as ‘Cyanobacteria’ and as ‘Non-Cyanobacteria’ respectively. **B** Non- metric multidimensional scaling (NMDS) plot based on Bray–Curtis distances of taxonomic composition of diazotroph communities. Symbols indicate diazotroph communities from different MSC fractions. Sampling stations are colored based on the distance to the Tonga volcanic arc (mesotrophic stations), i.e., darker colors indicate samples located closer to the arc area than lighter colors.

We further assessed the fate of the exported diazotroph community by collecting fresh particles in mesopelagic waters using the MSC. The diazotroph community composition (based on *nifH* amplicon sequencing) in the fast-sinking fraction at stations S05M and S10M generally mirrored that of traps with a dominance of sequences affiliated with *Trichodesmium* and ‘unknown NCD1’ (Fig. 5A). This was also the case at stations S03, S04 and S11, but not at stations S01, S02 and S08, where sequences assigned to *Candidatus* Atelocyanobacterium thalassa generally dominated the reads associated with cyanobacterial ASVs, consistent with the high abundances of UCYN-A1 found in the photic layer at those stations (Fig. S4). Community composition analysis confirmed this trend and showed that diazotrophic communities followed a spatial gradient largely influenced by the proximity to the Tonga archipelago (Fig. 5B), i.e., diazotrophic communities detected close to Tonga (mesotrophic waters) showed higher similarity to one another than to samples collected further away. As in sediment traps, the majority of *nifH* sequences were affiliated to non-cyanobacterial diazotrophs in MSC samples, whose composition was overall consistent with that of the traps, although some classes of Alpha-, Gamma- and Epsilon-proteobacteria not detected in sediment traps were present in the suspended and slow sinking MSC fractions (*Azorhizobium, Novosphingobium, Oceanobacter, Stenotrophomonas, Sulfurovum*).

As in sediment traps, five diazotroph groups were quantified by qPCR in the suspended, slow and fast-sinking fractions to more finely assess their sinking dynamics over short time scales (h). Overall, UCYN-A1 and *Trichodesmium* were the most abundant groups in the mesopelagic MSC samples (170 m to 1000 m) (Fig. S4). The MSC suspended fraction accounted for the most abundant pool of diazotrophs at all stations, followed by either the slow or the fast-sinking pools, depending on groups (Fig. S4a–e). At the mooring stations S05M and S10M, the total concentration of diazotrophs and the diazotroph community composition remained generally constant at the three sampled depths (170 m, 270 m and 1000 m), consistent with the results observed in sediment traps. To evaluate the export potential of each individual diazotroph group, we calculated the ratio of sinking versus non-sinking cells (the sum of the slow and fast-sinking fractions over the total). The proportion of sinking cells varied widely across the transect, but was the highest for UCYN-A1 (32 ± 33%), UCYN-C (31 ± 38%), Gamma-A (29 ± 19%) and UCYN-B (27 ± 11%), and the lowest for *Trichodesmium* (18 ± 20%) (Fig. S4f). Among the sinking fractions, UCYN-B and Gamma-A were generally equally distributed among the slow and fast-sinking fractions, whereas the majority of *nifH* gene copies were found in the fast-sinking fraction for UCYN-A1 (59%), UCYN-C (62%) and *Trichodesmium* (67%). The presence of Gamma-A in different sinking fractions might be due to the fact that Gamma-A could be attached to particles with different sizes [46] and, therefore, with different sinking velocities. Taken together, these results indicate that over short time scales (2 h, the conventional settling of particles following MSC deployment [29]): i) small UCYN (few µm) generally sink more efficiently than large *Trichodesmium* (>100 µm), ii) when sinking, *Trichodesmium* sinks rapidly, iii) UCYN-A1 and UCYN-C sink faster than UCYN-B and Gamma-A. A detailed imaging study performed on 170 m MSC samples at stations S05M and S10M indicates that 50–80% of phycoerythrin-containing UCYN in the fast-sinking fraction were organized into aggregates of tens to >250 cells measuring 30 to >100 µm, while the majority (60–95%) of UCYN were free living in the suspended fraction (Fig. S5). This indicates that under minimum turbulent agitation, only UCYN packaged into aggregates were large/dense enough to sink.

Concentrations of PON averaged across all stations 1.85 ± 0.49 µg L^−1^, 0.25 ± 0.13 µg L^−1^ and 0.26 ± 0.08 µg L^−1^ within the suspended, slow sinking and fast-sinking fractions (Fig. S6a). PON in the fast-sinking fraction contributed 11 ± 2%, while the slow sinking and suspended fractions contributed 11 ± 4% and 79 ± 5% in terms of total PON. Thus, ~22% of the PON was sinking out of the upper part of the MSC within 2 h during our study. We converted transparent expolymeric particles into C (TEP-C), that revealed higher concentrations in the suspended fraction compared to the fast-sinking fraction at all stations (TEP-C concentrations were null in the SS fraction) (Fig. S6b). The TEP-C:POC ratio was generally higher in the suspended fraction compared to the fast-sinking fraction. This indicates that, despite UCYN cells being embedded in TEP in FS samples, TEP were a minor contributor of the POC pool in the FS fraction.

Finally, we quantified diazotrophs (*nifH*) on vertical profiles spanning the water column between 200 m and 2000 m by using a Bottle-net. Overall, bottle-net tows confirm that diazotrophs are consistently present in this deep ocean layer, with concentrations averaging 1.4 × 10^7^ *nifH* gene copies m^−2^. Among the groups targeted by qPCR, the community was primarily dominated by UCYN-A1 (63% on average over all sampled stations) and *Trichodesmium* (27%), and secondarily by UCYN-B (9%) (Fig. S7), generally mirroring the diazotroph community in surface water (these numbers are conservative and may be underestimated as some individual UCYN pass through the 20 µm mesh net of the bottle-net). The 2000–200 m stock was relatively constant among stations, except at the most oligotrophic station S08, where it was lower by two orders of magnitude than that of other stations.

### Beyond subtropical south Pacific waters

**Fig. 6:**
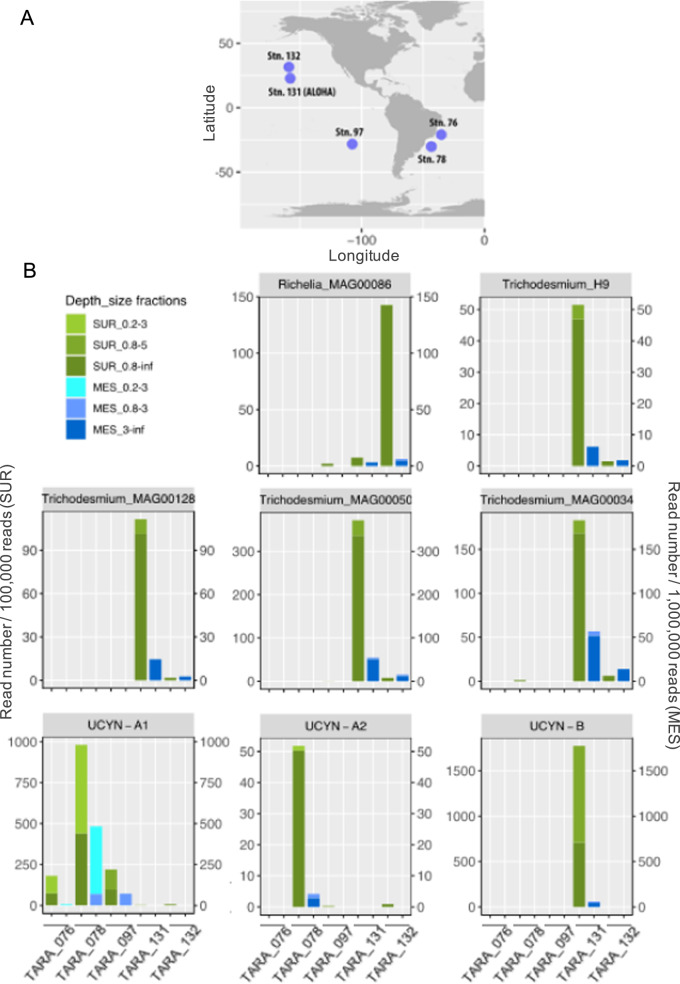
Distribution of diazotrophic cyanobacteria in surface and mesopelagic waters during the Tara Oceans expedition. **A** Geographical location of the *Tara* oceans stations used in this study. Only stations in which metagenomic reads belonging to *nifH* gene sequences from distinct cyanobacterial diazotrophs were present in surface and metagenomes were available both from surface and mesopelagic waters were selected. **B** Abundance of metagenomic reads recruited against different diazotrophic cyanobacteria genomes in surface and mesopelagic samples. See Table S3 for the complete dataset. Note that to better visualize the data, read abundance was expressed as number of reads per 100,000 total reads for surface samples (left axis) and per 1,000,000 total reads for mesopelagic samples (right axis).

To assess whether the sinking of globally distributed diazotrophs down to mesopelagic waters is a widespread feature, we explored the presence of diazotrophic cyanobacterial genomes using *Tara* Oceans metagenomes collected from other ocean basins [37, 47]. Recruitment of metagenomic reads against representative genomes of the diazotrophic community (*Trichodesmium, Richelia*, UCYN-B and UCYN-A1 and UCYN-A2; Table S4) across different size-fractions in surface and mesopelagic waters show that diazotrophs in general were systematically detected in mesopelagic waters at all five stations (Fig. 6; Table S4). As in the subtropical South Pacific Ocean, every specific group was detected at depth when present in surface, except when abundances in surface were very low (<6.7 reads/total 100,000 reads). In agreement with their cell size, *Trichodesmium*, *Richelia*, UCYN-B and UCYN-A2 reads were recovered in the size fraction >3 µm at mesopelagic depths, whereas the UCYN-A1 symbiosis was recovered in the 0.2–3 µm and 0.8–3 µm size fractions. Overall, this metagenomic analysis shows that the presence of diazotrophs in mesopelagic waters is not restricted to the subtropical South Pacific Ocean, suggesting that the transport of diazotrophs to the deep ocean is a widespread phenomenon in the tropical ocean.

### Potential biogeochemical implications

Excluding silicified DDAs [8], diazotrophs have seldom been regarded as important contributors to organic matter export. Yet, our results provide clear evidence that they are present and ubiquitous in the mesopelagic ocean and that the varying diazotroph morphologies (or groups) illustrate different behaviors.

We showed that diazotroph cell size is not necessarily a key variable controlling the diazotrophs’ ability to sink out of the euphotic zone. Small UCYN (1–8 µm) displayed the highest export turn-over rates (~10^−3^d^−1^), in the range, or higher than those reported for ballasted phytoplankton groups such as diatoms and coccolithophores [48]. Within UCYN, UCYN-A1 dominated the diazotroph community in mesopelagic waters of the subtropical South Pacific Ocean. Although the UCYN-A1 symbiosis has been detected once in mesopelagic waters of the South Atlantic Ocean [37, 49], their capacity to leave the photic layer and sink throughout the mesopelagic zone down to 1000 m (Figs. 3, 6) had not been thoroughly explored before. As UCYN-A rank among the most abundant diazotrophs in the global ocean [13] and span from tropical to polar waters [50], we suggest that they potentially contribute significantly to ocean organic matter sequestration. Some UCYN-A ecotypes (e.g. UCYN-A2) live in obligate symbiosis with a calcified coccolithophore [51], hence their export is likely enhanced by this ballast mineral increasing the density of sinking particles to which they are associated. In future studies, better constrains on UCYN-A sedimentation rates and aggregation processes would be of primary importance to assess their role in particle flux and cycling.

UCYN-B and -C are free-living unicellular cyanobacteria and their export pathway is therefore independent from any mineral ballasted host. Our results indicate that the sinking of such small cells is made possible through the aggregation of UCYN into large-sized aggregates (30->100 µm) comprised of tens to hundreds of cells, large/dense enough to sink. Such aggregation can be promoted though multiple mechanisms including TEP-induced aggregation, but also ingestion by grazers [52, 53] and incorporation into large fecal pellets. In addition, UCYN-B produce large quantities of glycogen as an energy storage for N_2_ fixation during the night, that increases the density of cells, and may also contribute to explain their export out of the euphotic zone [54]. The presence of UCYN-B in sediment traps is in accordance with previous reports of *Crocosphaera watsonii* sequences at station ALOHA either just below the euphotic zone [23], or as deep as 4000 m [22]. The visualization of our sediment trap samples shows that they can be subsequently embedded in mixed aggregates with other cells and debris (Figs. 4, S2). The UCYN-B large (>4 µm) ecotype, which dominated in this study (Fig. 4), produces large amounts of C-rich TEPs -at rates one to two order of magnitude higher than that of diatoms and coccolithophorids [55], probably in response to nutrient limitation and excess light [56]. In cultures of *Crocosphaera watsonii*, TEP account for ~22% of the particulate C pool [55]. Hence, TEP produced by UCYN-B not only provides a matrix for the formation of large aggregates, but may also account for a significant fraction of C export. TEP are indeed greatly enriched in C relative to N (C:N ~25 [55]).

*Trichodesmium* was generally the second contributor to the diazotroph community targeted by qPCR in mesopelagic waters, which contradicts the common assertion that they are remineralized in the euphotic layer [4, 14]. Several potential mechanisms could explain the presence of *Trichodesmium* in mesopelagic waters. *Trichodesmium* colonies can migrate vertically to exploit the deep phosphate stock [57] (the phosphacline was around 200 m in our study region at the time of the cruise). According to this theory, they overcome their positive buoyancy by fixing C that results in carbohydrate ballasting. However, Walsby [58] observed that 100% of the gas vacuoles of *Trichodesmium erythraeum* (the most abundant species in this study) collapse at depths between 105 m and 120 m, resulting in a loss of their buoyancy. Hence, *Trichodesmium* could be locked in a persistent and irreversible downward trajectory. Alternatively, Berman-Frank et al. [59] have shown that programmed cell death induces internal cellular degradation, gas-vacuole loss and increased production of TEP, also resulting in an increase in the vertical flux of *Trichodesmium* [60]. Whatever the mechanism behind the vertical flux of *Trichodesmium*, we report here higher abundances at 1000 m compared to those at shallower depths at both stations, consistent with microscopic observations showing intact colonies at 1000 m. This likely results from a spatiotemporal decoupling between production and export [61]. Despite sinking less efficiently than UCYN in the aggregates, *Trichodesmium* are much larger and contain more C and N per filament than UCYN [13], and may account for a significant fraction of the PON export.

Based on diazotroph abundances and published PON content per diazotroph (see Methods), we estimated that diazotrophs would contribute between 1% at 170 m to ~85% at 1000 m of PON export at Station S05M and between ~1% (170 m and 270 m) to 2% at station S10M, with *Trichodesmium* being the major contributor (Table S2). These estimates provide a first approximation and must be considered with caution as we used several assumptions in our calculations (see Methods) and we have only taken into account the diazotrophs quantified in the traps (mostly cyanobacteria). Although non-cyanobacterial diazotrophs dominated in exported material (59–71% of sequences), their contribution to export was not taken into account here as their C and N composition remains unknown. Thus, our assessment may actually underestimate the contribution of diazotrophs to overall PON export in this study.

More than 90% of the organic matter sinking below the euphotic zone is respired before it reaches a depth of 1000 m [9]. Fast-sinking particles will therefore theoretically make a greater contribution to the deep ocean flux than slow sinking particles since the latter will be rapidly recycled at shallow depths. The relative similarity of the taxonomic diazotroph community composition in mesopelagic waters, sampled by three independent sampling approaches (traps, bottle-net, and MSC), compared to the diazotrophic composition of the euphotic layer suggests a rapid export mode of diazotrophs. This is further confirmed by i) the high proportion of diazotroph groups quantified in the MSC fast-sinking fraction, ii) the whole cells and colonies and high Chlorophyll *a*: phaeopigments ratios in sediment traps, indicative of undegraded phytoplankton (Fig. 4; Fig. S2). Finally, iii) while PON export fluxes were attenuated with depth in our study, which is a classical feature in the oligotrophic ocean [62], the diazotroph export fluxes were not, suggesting a high transfer efficiency to the deep ocean, and thus high enough sinking velocities that would escape short-term remineralization. Indeed, a recent study performed during the same cruise [63] reveals significant N_2_ fixation rates in sediment trap samples, suggesting that part of the diazotroph community in mesopelagic waters sunk fast enough to remain alive at mesopelagic depths. Indeed, they report *Trichodesmium* sp. sinking velocities of ~300 md^−1^, in accordance with those measured by Bar-Zeev et al. [60] (~200 md^−1^). We calculated that *Trichodesmium* sinking at those rates (200–300 md^−1^) would take 3 to 5 days to reach 1000 m, which would be compatible with finding active cells observed at those depths [63].

## Conclusions

Our findings challenge the common assumption that the fate of diazotroph-derived production is constrained to the surface ocean. Further, our results redefine the role of diazotrophs by establishing their significant contribution to global carbon cycling via the biological carbon pump. Moreover, we provide new insights into the group-specific export of diazotrophs in the ocean, revealing a previously-unquantified contribution of UCYN and *Trichodesmium* to the overall export fluxes. A mechanistic understanding of particle formation, aggregation, sinking velocity of diverse diazotrophs is needed to integrate them into models and improve the accuracy of current regional and global estimates of export. Additionally, as diazotroph distribution is not restricted to the tropical ocean, future research is needed to explore the direct gravitational export of diazotrophs in temperate and polar waters.

Direct export through gravitational settling of diazotrophs is most likely supplemented by diazotroph export through secondary pathways. Diazotrophs release in seawater 10–50% of recently fixed N_2_ (referred to as Diazotroph Derived, DDN) as NH_4_^+^ and dissolved organic N (DON) [64]. This DDN is potentially available for assimilation by the surrounding phytoplanktonic communities, supporting their growth and leading a potential secondary (indirect) export pathway of diazotroph-derived particulate organic matter [7, 65]. Moreover, diazotrophs are also grazed by zooplankton [53], which packages diazotroph organic matter into fecal pellets that in turn, sink rapidly [65] and might play a major role in DDN export to the deep ocean. New approaches to decipher diazotroph export pathways (direct *vs* indirect) are required in future studies if we are to fully comprehend the role of N_2_ fixation in the biological carbon (and nitrogen) pump. This is an especially pressing question given that current climate models predict an expansion of the oligotrophic ocean (60% of our oceans) [66], where diazotrophs thrive. N_2_ fixation will thus likely be crucial to supporting primary productivity and export in the future ocean.

## Supplementary information

Supplemental matrerial

Table S1

Table S2

Table S3

Table S4

## Data Availability

Data are available in the main text or the supplementary materials. Sequences have been deposited to NCBI with accession numbers SAMN24579080-SAMN24579145.
