# Supplementary material for: Diazotrophs are overlooked contributors to carbon and nitrogen export to the deep ocean
Source: ISME J. 2022 Sep 26;17(1):47–58. doi: 10.1038/s41396-022-01319-3 (PMC9750961; doi:10.1038/s41396-022-01319-3)
Supplement: Supplementary file 1 — Supplemental matrerial [file 41396_2022_1319_MOESM1_ESM.pdf]

Supplementary information

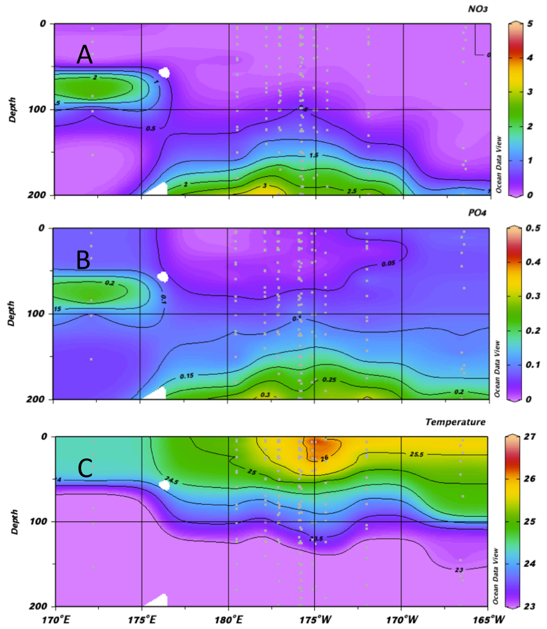

Fig. S1. Horizontal and vertical distributions of A. nitrate concentrations (μmol L^{-1}), B. phosphate concentrations (μmol L^{-1}) and C. Seawater temperature (°C) across the TONGA transect. Y axis: pressure (dbar), X axis: longitude; grey dots correspond to sampling depths at the various stations.

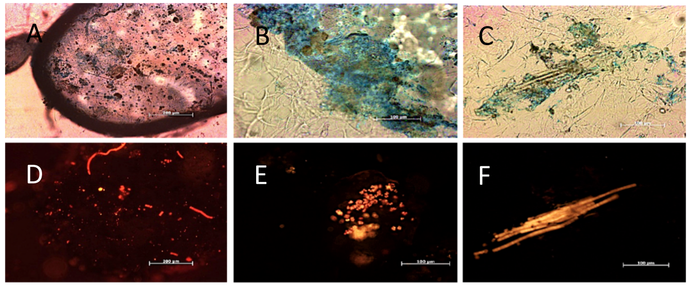

Fig. S2. Microscopy images showing examples of diazotrophs embeded in TEP in sediment trap samples collected at 170 m (A,D), 270 m (B,E), and 1000 m (C,F) at station S10M. Each trio of slides shows an identical image viewed with a-c. white light (upper panels) to highlight staining by Alcian Blue for transparent exopolymeric particles (TEP) seen in blue shades, and d-f. Phycoerythrin filter (lower panels). Both panels illustrate the organic matrix surrounding many of the particles in the traps. Visualized on the left panels are phycoerythrin containing cyanobacteria identified as *Trichodesmium* at 1000 m, *Crocosphaera*- and *Synechoccocus*-like ecotypes at 270 m and a combination of all at 170m. Slides are representative images from these depths. All three traps contained each of the described organisms.

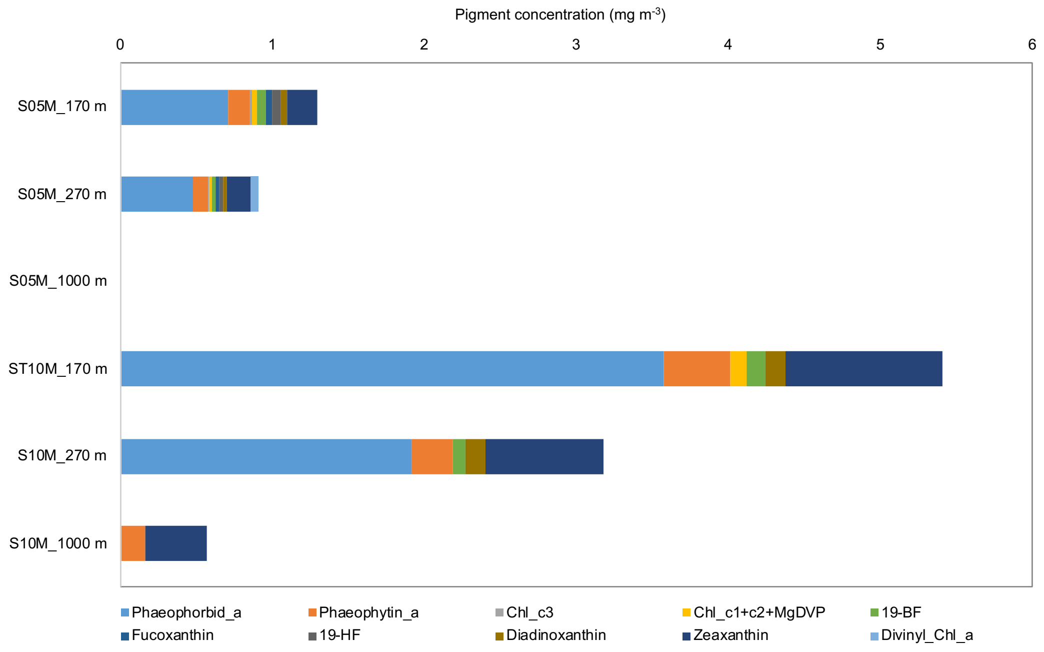

Fig. S3. Pigment concentrations (mg m^{-3}) in sediment trap samples at stations S05M and S10M. No data are available at station S05M 1000 m.

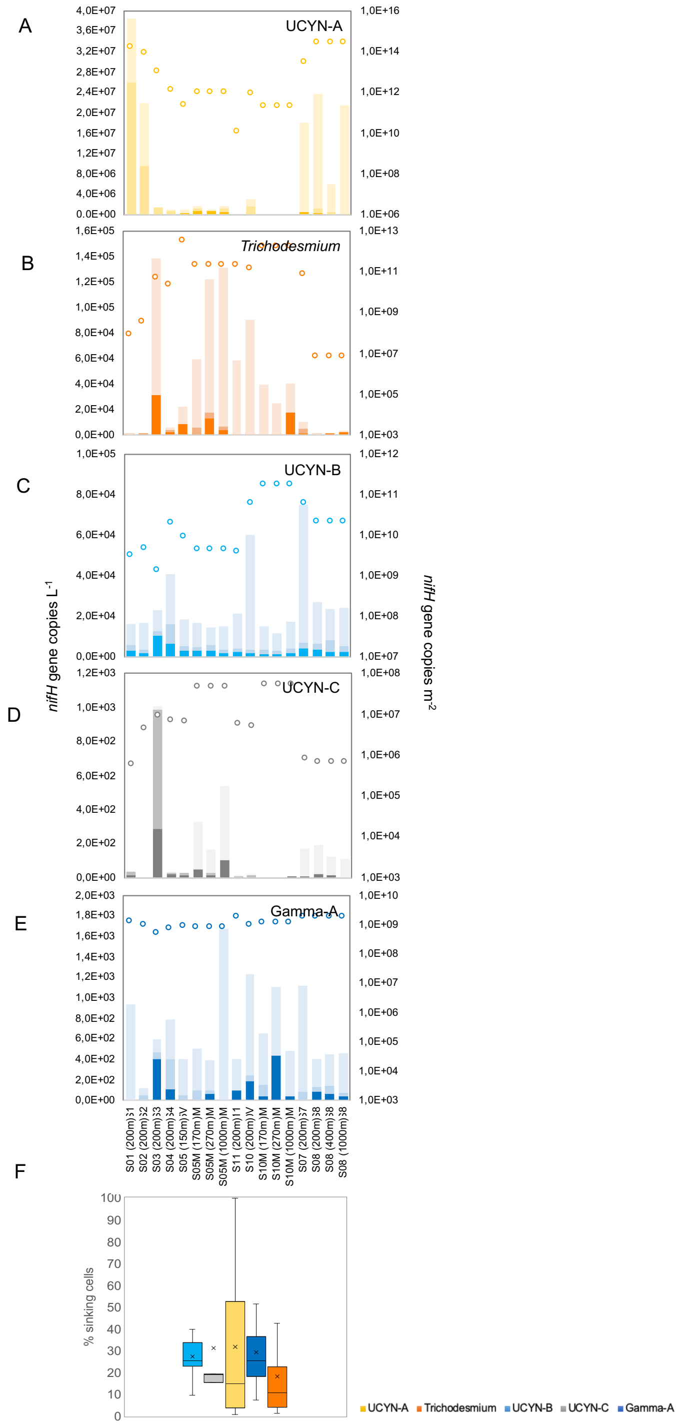

Figure S4. A-E. Quantification of the five diazotroph groups targeted by qPCR in Marine Snow catcher samples in nifH gene copies L^{-1}: UCYN-A1 symbiosis, *Trichodesmium*, UCYN-B, UCYN-C, and Gamma-A in the suspended (light color), slow sinking (medium color) and fast sinking (dark color) pools of the MSC samples across the transect (left Y axis, *nifH* gene copies L^{-1}). Dots represent the integrated abundances of each phylotype (right Y axis, *nifH* gene copies m^{-2}) in the photic (~0-100 m) layer. F. Percentage of sinking versus non-sinking diazotrophs for each group.

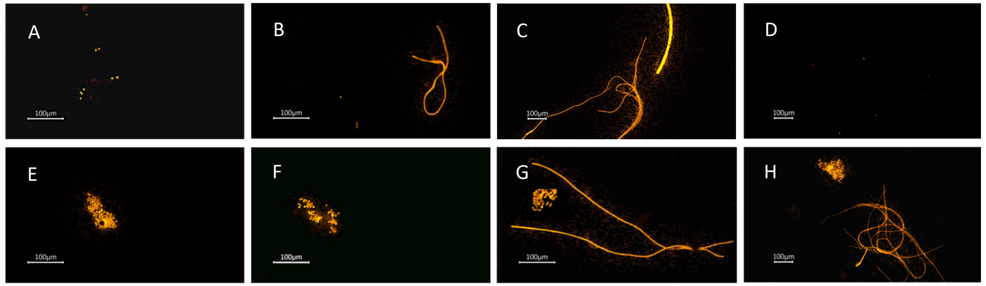

Fig. S5. Epifluorescence microscopy images showing typical examples of phycoerythrin-containing UCYN in the suspended (A-D) and fast sinking (E-H) fractions of the MSC (S05M and S10M, 170 m).

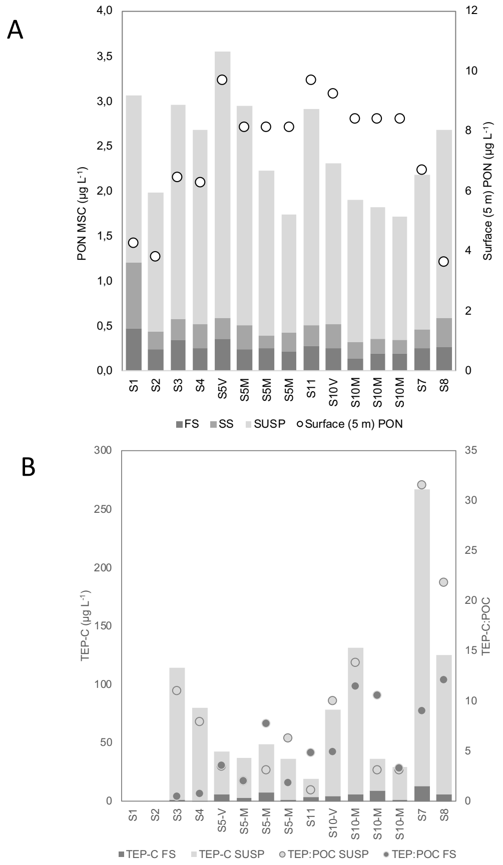

Fig. S6. A. PN concentrations (µg L^{-1}) of the suspended, slow and fast sinking pools of the MSC across the transect. Dots represent the surface (5 m) PN pool. B. TEP-C concentrations (µg L^{-1}) in the suspended and fast sinking pools. Dots represent the TEP-C:POC ratio for both fractions (suspended: light grey, and fast sinking: dark grey).

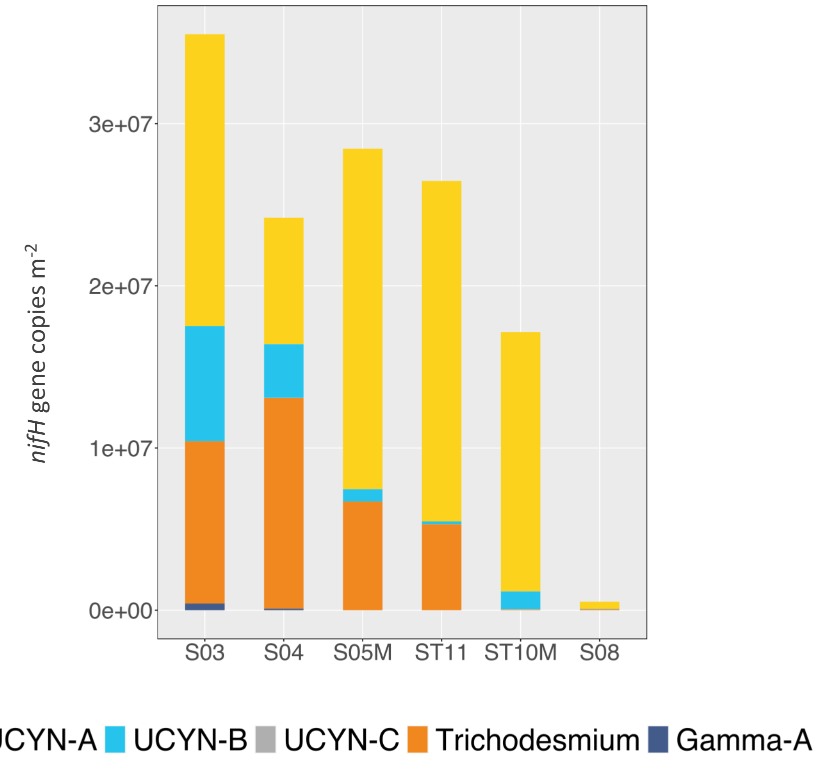

Figure S7. Abundance (*nifH* gene copies m^{-2}) of the five diazotroph groups targeted by qPCR (UCYN-A1 symbiosis, *Trichodesmium*, UCYN-B, UCYN-C, and Gamma-A) in the bottle-net samples across the transect, representing an integrated sample from 200 m to 2000 m.

Table S1. ASV annotations table (.xls file)

Table S2. Estimation of the contribution of diazotrophs to PON export at stations S05M and S10M at 170 m, 270 m and 1000 m.

				Potential contribution of diazotrophs to PN export flux (%)					
	Published C content per cell*	C:N**	Calculated N content per cell	Station S05M			Station S10M		
	pg C cell-1	-	pg N cell-1	170 m	270 m	1000 m	170 m	270 m	1000 m
PN export flux (mg N m-2 d-1)	-	-	-	4.9±0.6	6.1±1.2	1.6±0.2	13.4±1.3	9.7±0.6	5.2±0.1
UCYN-A1	0.1-0.3 (0.2)	6.3	0.02-0.05 (0.03)	0.2-0.5 (0.3)	0.7-2 (1)	11-27 (16)	0.2-0.6 (0.3)	0.2-0.6 (0.4)	0.5-1 (1)
UCYN-B	4-50 (20)	6	0.1-0.6 (0.2)	0.1-0.5 (0.2)	0.1-1.2 (0.5)	2-16 (6)	0.1-0.5 (0.2)	0.0-0.5 (0.2)	0.0-0.4 (0.2)
UCYN-C	5-24 (10)	8.5	0.6-3 (1)	0.01-0.02 (0.01)	0.00-0.01 (0.00)	0.00-0.02 (0.01)	0.00-0.01 (0.00)	0.00-0.01 (0.00)	0.00-0.01 (0.01)
Gamma A	0,0124	-	0.001-0.003 (0.002)	<0,001	<0,001	<0,001	<0,001	<0,001	<0,001
Trichodesmium spp.	42-160 (65)	6	67-27 (11)	0.4-1.4 (0.6)	1.3-4.9 (2)	44-69 (68)	0	0	0.7-2.7 (1.1)
Sum of diazotrophs	-	-	-	*1-3 (1)*	*2-7 (3)*	*55-96 (85)*	*0.3-1.1 (0.6)*	*0.3-1.2 (0.6)*	*1.3-4.4 (2)*

**From Luo et al., (2012) for UCYN-A, UCYN-B, UCYN-B and Trichodesmium. From Fukuda et al., (1998) for Gamma-A*

***From Martinez-Perez et al., (2016) for UCYN-A; From Knapp et al., (2012) for UCYN-B and Trichodesmium; From Berthelot et al., (2015) for UCYN-C; From Fukuda et al., (1998) for Gamma-A*

Table S3. Diazotroph genomes used as reference for metagenomic recruitement

Organism	Genome Accession	References
Crocosphaera watsonii WH8502	CAQK01000001 - CAQK01000869	https://doi.org/10.1111/jpy.12090
Richelia_TARA_PON_109_MAG_00086	https://figshare.com/articles/dataset/Marine_diazotrophs/14248283	https://doi.org/10.1101/2021.03.24.436778
Trichodesmium thiebautii H9-4	SAMN03421272	https://doi.org/10.1073/pnas.1422332112
Trichodesmium erythraeum IMS101	SAMN02598485	https://doi.org/10.1073/pnas.1422332112
Trichodesmium_TARA_AON_82_MAG_00128	https://figshare.com/articles/dataset/Marine_diazotrophs/14248283	https://doi.org/10.1101/2021.03.24.436778
Trichodesmium_TARA_IOS_50_MAG_00050	https://figshare.com/articles/dataset/Marine_diazotrophs/14248283	https://doi.org/10.1101/2021.03.24.436778
Trichodesmium_TARA_PON_109_MAG_00034	https://figshare.com/articles/dataset/Marine_diazotrophs/14248283	https://doi.org/10.1101/2021.03.24.436778
UCYN-A1	NC_013771.1	https://doi.org/10.1038/nature08786
UCYN-A2	JPSP01000000	https://doi.org/10.1038/ismej.2014.167

Table S4. Description of Tara Oceans samples used in this study and number of recruited reads per station and size fraction for each diazotroph genomes used as reference (.xls file)
